# Association of Increased Frequencies of HLA-DPB1*05∶01 with the Presence of Anti-Ro/SS-A and Anti-La/SS-B Antibodies in Japanese Rheumatoid Arthritis and Systemic Lupus Erythematosus Patients

**DOI:** 10.1371/journal.pone.0053910

**Published:** 2013-01-08

**Authors:** Hiroshi Furukawa, Shomi Oka, Kota Shimada, Shoji Sugii, Atsushi Hashimoto, Akiko Komiya, Naoshi Fukui, Tatsuo Nagai, Shunsei Hirohata, Keigo Setoguchi, Akira Okamoto, Noriyuki Chiba, Eiichi Suematsu, Taiichiro Miyashita, Kiyoshi Migita, Akiko Suda, Shouhei Nagaoka, Naoyuki Tsuchiya, Shigeto Tohma

**Affiliations:** 1 Clinical Research Center for Allergy and Rheumatology, Sagamihara Hospital, National Hospital Organization, Sagamihara, Japan; 2 Department of Rheumatology, Tokyo Metropolitan Tama Medical Center, Fuchu, Japan; 3 Department of Rheumatology, Sagamihara Hospital, National Hospital Organization, Sagamihara, Japan; 4 Department of Rheumatology and Infectious Disease, Kitasato University School of Medicine, Sagamihara, Japan; 5 Allergy and Immunological Diseases, Tokyo Metropolitan Cancer and Infectious Diseases Center Komagome Hospital, Tokyo, Japan; 6 Department of Rheumatology, Himeji Medical Center, National Hospital Organization, Himeji, Japan; 7 Department of Rheumatology, Morioka Hospital, National Hospital Organization, Morioka, Japan; 8 Department of Internal Medicine and Rheumatology, Clinical Research Institute, National Hospital Organization, Kyushu Medical Center, Fukuoka, Japan; 9 Nagasaki Medical Center, National Hospital Organization, Omura, Japan; 10 Department of Rheumatology, Yokohama Minami Kyosai Hospital, Yokohama, Japan; 11 Molecular and Genetic Epidemiology Laboratory, Faculty of Medicine, University of Tsukuba, Tsukuba, Japan; University of Bergen, Norway

## Abstract

**Introduction:**

Autoantibodies to ribonucleoprotein are associated with a variety of autoimmune diseases, including rheumatoid arthritis (RA). Many studies on associations between human leukocyte antigen (HLA) alleles and RA have been reported, but few have been validated in RA subpopulations with anti-La/SS-B or anti-Ro/SS-A antibodies. Here, we investigated associations of *HLA class II* alleles with the presence of anti-Ro/SS-A or anti-La/SS-B antibodies in RA.

**Methods:**

An association study was conducted for *HLA-DRB1, DQB1,* and *DPB1* in Japanese RA and systemic lupus erythematosus (SLE) patients that were positive or negative for anti-Ro/SS-A and/or anti-La/SS-B antibodies.

**Results:**

An increased prevalence of certain class II alleles was associated with the presence of anti-Ro/SS-A antibodies as follows: DRB1*08∶03 (*Pc* = 3.79×10^−5^, odds ratio [OR] 3.06, 95% confidence interval [CI] 1.98–4.73), DQB1*06∶01 (*Pc* = 0.0106, OR 1.70, 95%CI 1.26–2.31), and DPB1*05∶01 (*Pc* = 0.0040, OR 1.55, 95%CI 1.23–1.96). On the other hand, DRB1*15∶01 (*Pc* = 0.0470, OR 3.14, 95%CI 1.63–6.05), DQB1*06∶02 (*Pc* = 0.0252, OR 3.14, 95%CI 1.63–6.05), and DPB1*05∶01 (*Pc* = 0.0069, OR 2.27, 95% CI 1.44–3.57) were associated with anti-La/SS-B antibodies. The DPB1*05∶01 allele was associated with anti-Ro/SS-A (*Pc* = 0.0408, OR 1.69, 95% CI 1.19–2.41) and anti-La/SS-B antibodies (*Pc* = 2.48×10^−5^, OR 3.31, 95%CI 2.02–5.43) in SLE patients.

**Conclusion:**

HLA-DPB1*05∶01 was the only allele associated with the presence of both anti-Ro/SS-A and anti-La/SS-B antibodies in Japanese RA and SLE patients.

## Introduction

Rheumatoid arthritis (RA) is a chronic systemic inflammatory disease susceptibility to which is associated with genetic and environmental factors [1], [2], [3]. Altered frequencies of human leukocyte antigen (HLA) alleles are known to be associated with RA in most ethnic groups studied. Some *HLA-DR* alleles are reported to be positively associated with RA susceptibility [4]. A conserved amino acid sequence at position 70–74 (QKRAA, RRRAA, or QRRAA) in the HLA-DRβ chain is shared between the RA-associated *HLA-DR* alleles; this was therefore designated the shared epitope (SE) [4].

The presence of autoantibodies to ribonucleoprotein is associated with a variety of autoimmune diseases, including Sjögren’s Syndrome (SS), systemic lupus erythematosus (SLE), and RA. Anti-La/SS-B antibodies share many features with anti-Ro/SS-A antibodies, and almost all anti-La/SS-B antibody-positive RA patients also have anti-Ro/SS-A antibodies, whereas about one fifth of anti-Ro/SS-A antibody-positive RA patients also have anti-La/SS-B antibodies. HLA-DR2 (DRB1*15 and *16) and DR3 are strongly associated with anti- Ro/SS-A or anti-La/SS-B antibodies in European primary SS populations [5], [6], [7], [8]. On the other hand, DRB1*08∶03 was reported to be associated with anti-La/SS-B antibodies and *15∶01 with anti-Ro/SS-A antibodies in primary SS, SLE, and asymptomatic individuals in the Japanese population [9]. However, few studies have focused on the association of anti-La/SS-B and anti-Ro/SS-A antibodies with HLA alleles in RA [10]. Here, we elucidate *HLA class II* associations with the presence of autoantibody in Japanese RA patients.

## Materials and Methods

### Patients and Controls

Nine hundred twenty five RA and 622 SLE patients were recruited at Sagamihara Hospital, Nagasaki Medical Center, Yokohama Minami Kyosai Hospital, Tama Medical Center, Kitasato University, Komagome Hospital, Himeji Medical Center, Morioka Hospital, and Kyushu Medical Center. All patients were native Japanese living in Japan. All patients with RA fulfilled the 1988 American College of Rheumatology Criteria for RA [11] and did not overlap any other collagen diseases. All patients with SLE fulfilled the American College of Rheumatology criteria for SLE [12]. The RA patients with SS also fulfilled the Japanese Ministry of Health Criteria for the diagnosis of SS [13]. This study was reviewed and approved by the research ethics committees of each participating institute, Sagamihara Hospital Research Ethics Committee, Nagasaki Medical Center Research Ethics Committee, Yokohama Minami Kyosai Hospital Research Ethics Committee, Tama Medical Center Research Ethics Committee, University of Tsukuba Research Ethics Committee, Kitasato University Ethics Committee, Komagome Hospital Ethics Committee, Himeji Medical Center Ethics Committee, Morioka Hospital Ethics Committee, and Kyushu Medical Center Ethics Committee. Written informed consent was obtained from all study participants. This study was conducted in accordance with the principles expressed in the Declaration of Helsinki. Anti-Ro/SS-A and anti-La/SS-B antibodies were detected using Mesacup-2 test (Medical & Biological Laboratories, Nagoya, Japan), or Ouchterlony double immunodiffusion method (TFB, Hachioji, Japan). RA patients who visited Sagamihara Hospital (n = 1538) were classified as anti-Ro/SS-A antibodies positive RA (n = 225, 14.6%) and anti-La/SS-B antibodies positive RA (n = 37, 2.4%).

### Genotyping

Genotyping of *HLA-DRB1, DQB1*, and *DPB1* was performed by polymerase chain reaction using sequence-specific oligonucleotide probes, WAKFlow HLA typing kits (Wakunaga, Hiroshima, Japan), using a Bio-Plex 200 system (Bio-Rad, Hercules, CA). *HLA-DRB1* alleles encoding the SE are as follows: *01∶01, *04∶01, *04∶04, *04∶05, *04∶10, *10∶01, *14∶02, and *14∶06 [14]. One of each *DRB1* and *DQB1* locus could not be typed in the present study. These were revealed to be novel HLA alleles, DRB1* 08∶36∶02 and DQB1*06∶51, by sequencing of the isolated alleles [15].

### Statistical Analysis

Differences of RA characteristics, allele frequencies, or amino acid residue frequencies were analyzed by Student’s t-test or Fisher’s exact test using 2×2 contingency tables. Adjustment for multiple comparisons was performed using the Bonferroni method. Corrected *P* (*Pc*) values were calculated by multiplying the *P* value by the number of alleles or amino acid residues tested.

## Results

### Characteristics of Anti-Ro/SS-A and/or Anti-La/SS-B Antibody-positive RA and SLE Patients

Characteristics of anti-Ro/SS-A-positive but anti-La/SS-B-negative [Ro(+)La(−)] RA and anti-Ro/SS-A- and anti-La/SS-B-positive [Ro(+)La(+)] RA patients are given in Table 1. Mean age and percentage of males in the Ro(+)La(−)RA and Ro(+)La(+)RA groups were lower than in the anti-Ro/SS-A- and anti-La/SS-B-negative [Ro(−)La(−)] patients. Percentage of secondary SS in the Ro(+)La(−)RA and Ro(+)La(+)RA was higher than in the Ro(−)La(−)RA. There were no significant differences in terms of disease duration, rheumatoid factor or anti-citrullinated peptide antibody positivity, or Steinbrocker stage.

**Table 1:** Characteristics of RA and SLE patients.

	Ro(+)La(−)RA	Ro(+)La(+)RA	Ro(−)La(−)RA	*P* [Ro(+)La(−) vs. Ro(−)La(−)]	*P* [Ro(+)La(+) vs. Ro(−)La(−)]
Number	181	40	704		
Mean age, years (SD)	59.8 (13.1)	59.6 (12.2)	63.9 (11.8)	0.0002*	0.0390*
Male, n (%)	15 (8.3)	0 (0.0)	143 (20.5)	0.0001	0.0003
Disease duration, years (SD)	14.3 (10.3)	17.7 (13.5)	14.3 (10.9)	0.9940*	0.1017*
Steinbrocker stage III and IV, n (%)	75 (51.0)	18 (62.1)	311 (57.4)	0.1897	0.7019
Association of secondary SS, n (%)	34 (18.8)	12 (30.0)	26 (3.7)	1.41×10^−10^	1.07×10^−7^
Rheumatoid factor positive, n (%)	146 (89.0)	30 (88.2)	545 (87.6)	0.6878	1.0000
ACPA positive, n (%)	135 (87.1)	30 (96.8)	506 (91.8)	0.0832	0.4999
	**Ro(+)La(−)SLE**	**Ro(+)La(+)SLE**	**Ro(−)La(−)SLE**	***P*** ** [Ro(+)La(−) vs. Ro(−)La(−)]**	***P*** ** [Ro(+)La(+) vs. Ro(−)La(−)]**
Number	129	45	137		
Mean age, years (SD)	49.1 (15.3)	46.0 (15.5)	50.0 (14.7)	0.7118*	0.1676*
Male, n (%)	11 (8.5)	2 (4.4)	15 (10.9)	0.5417	0.2483
Disease duration, years (SD)	13.3 (12.1)	7.2 (5.8)	15.6 (12.1)	0.1656*	0.0001*
Association of secondary SS, n (%)	6 (4.7)	3 (6.7)	1 (0.7)	0.0598	0.0473
Anti-dsDNA antibody positive, n (%)	104 (80.6)	31 (68.9)	104 (75.9)	0.3948	0.2720

SS: Sjögren’s syndrome, RA: rheumatoid arthritis, SLE: systemic lupus erythematosus, ACPA: anti-citrullinated peptide antibody, dsDNA: double-stranded-DNA, Ro(+)La(−): anti-Ro/SS-A-positive but anti-La/SS-B-negative, Ro(+)La(+): anti-Ro/SS-A- and anti-La/SS-B-positive, Ro(−)La(−): anti-Ro/SS-A- and anti-La/SS-B-negative. Association was tested by Fisher’s exact test using 2×2 contingency tables or Student’s t-test. *Student’s t-test was employed.

Characteristics of Ro(+)La(−) and Ro(+)La(+) SLE patients are also given in Table 1. Disease duration in the Ro(+)La(+) groups was shorter than in the Ro(−)La(−). Percentage of secondary SS in the Ro(+)La(+)SLE was higher than in the Ro(−)La(−)SLE. There were no significant differences in terms of mean age, percentage of males or anti-double-stranded-DNA antibody positivity.

### Association of HLA Class II Allele Frequencies with the Presence of Anti-Ro/SS-A Antibodies

We tested whether *HLA class II* was associated with the presence of anti-Ro/SS-A antibodies, comparing the Ro(+)La(−)RA and Ro(−)La(−)RA groups. A significant positive association was found for DRB1*08∶03 and anti-Ro/SS-A antibodies (*Pc* = 3.79×10^−5^, odds ratio [OR] 3.06, 95% confidence interval [CI] 1.98–4.73, Table 2). The DQB1*06∶01 allele was also associated with the presence of anti-Ro/SS-A antibodies (*Pc* = 0.0106, OR 1.70, 95% CI 1.26–2.31, Table 2). Further, the HLA-DPB1*05∶01 allele was associated with anti-Ro/SS-A antibodies (*Pc* = 0.0040, OR 1.55, 95% CI 1.23–1.96). Frequencies of DR4 and SE alleles were lower in Ro(+)La(−)RA than in Ro(−)La(−)RA (*P* = 0.0146, OR 0.73, 95% CI 0.57–0.94 and *P* = 0.0089, OR 0.73, 95% CI 0.57–0.92, respectively). Frequencies of DR2 alleles (DRB1*15 and *16) in Ro(+)La(−)RA and Ro(−)La(−)RA were not significantly different (*P* = 0.1028, OR 1.29). Thus, there were positive associations between certain alleles of *HLA-DRB1, DQB1,* and *DPB1* and the presence of anti-Ro/SS-A antibodies in RA patients.

**Table 2:** HLA allele frequencies in Ro(+)La(−) RA patients.

	Ro(+)La(−)	Ro(−)La(−)	*P*	OR	*Pc*	95%CI
DRB1*01∶01	21 (5.8)	110 (7.8)	0.2162	0.73	NS	
DRB1*04∶01	6 (1.7)	52 (3.7)	0.0667	0.44	NS	
DRB1*04∶03	5 (1.4)	20 (1.4)	1.0000	0.97	NS	
DRB1*04∶04	0 (0.0)	3 (0.2)	1.0000	0.55	NS	
DRB1*04∶05	92 (25.4)	406 (28.8)	0.2132	0.84	NS	
DRB1*04∶06	7 (1.9)	25 (1.8)	0.8256	1.09	NS	
DRB1*04∶07	0 (0.0)	3 (0.2)	1.0000	0.55	NS	
DRB1*04∶10	4 (1.1)	33 (2.3)	0.2136	0.47	NS	
DRB1*07∶01	0 (0.0)	6 (0.4)	0.6086	0.30	NS	
DRB1*08∶02	11 (3.0)	21 (1.5)	0.0727	2.07	NS	
DRB1*08∶03	38 (10.5)	52 (3.7)	1.35×10^−6^	3.06	3.79×10^−5^	(1.98–4.73)
DRB1*08∶23	1 (0.3)	0 (0.0)	0.2045	11.69	NS	
DRB1*09∶01	42 (11.6)	243 (17.3)	0.0082	0.63	0.2283	(0.44–0.89)
DRB1*10∶01	3 (0.8)	12 (0.9)	1.0000	0.97	NS	
DRB1*11∶01	3 (0.8)	17 (1.2)	0.7809	0.68	NS	
DRB1*12∶01	14 (3.9)	39 (2.8)	0.2985	1.41	NS	
DRB1*12∶02	7 (1.9)	20 (1.4)	0.4724	1.37	NS	
DRB1*13∶01	1 (0.3)	3 (0.2)	1.0000	1.30	NS	
DRB1*13∶02	7 (1.9)	60 (4.3)	0.0434	0.44	NS	(0.20–0.98)
DRB1*14∶02	0 (0.0)	2 (0.1)	1.0000	0.78	NS	
DRB1*14∶03	8 (2.2)	10 (0.7)	0.0180	3.16	0.5051	(1.24–8.06)
DRB1*14∶05	3 (0.8)	14 (1.0)	1.0000	0.83	NS	
DRB1*14∶06	9 (2.5)	15 (1.1)	0.0689	2.37	NS	
DRB1*14∶07	0 (0.0)	1 (0.1)	1.0000	1.29	NS	
DRB1*14∶54	13 (3.6)	34 (2.4)	0.2041	1.51	NS	
DRB1*15∶01	28 (7.7)	75 (5.3)	0.1006	1.49	NS	
DRB1*15∶02	36 (9.9)	124 (8.8)	0.5373	1.14	NS	
DRB1*16∶02	2 (0.6)	8 (0.6)	1.0000	0.97	NS	
DQB1*02∶01	0 (0.0)	6 (0.4)	0.6086	0.30	NS	
DQB1*03∶01	39 (10.8)	133 (9.4)	0.4280	1.16	NS	
DQB1*03∶02	22 (6.1)	73 (5.2)	0.5134	1.18	NS	
DQB1*03∶03	46 (12.7)	229 (16.3)	0.1038	0.75	NS	
DQB1*03∶06	0 (0.0)	3 (0.2)	1.0000	0.55	NS	
DQB1*04∶01	96 (26.5)	432 (30.7)	0.1384	0.82	NS	
DQB1*04∶02	7 (1.9)	35 (2.5)	0.6987	0.77	NS	
DQB1*05∶01	25 (6.9)	125 (8.9)	0.2461	0.76	NS	
DQB1*05∶02	14 (3.9)	27 (1.9)	0.0471	2.06	0.7071	(1.07–3.97)
DQB1*05∶03	6 (1.7)	29 (2.1)	0.8322	0.80	NS	
DQB1*06∶01	72 (19.9)	179 (12.7)	0.0007	1.70	0.0106	(1.26–2.31)
DQB1*06∶02	27 (7.5)	75 (5.3)	0.1291	1.43	NS	
DQB1*06∶03	1 (0.3)	3 (0.2)	1.0000	1.30	NS	
DQB1*06∶04	6 (1.7)	57 (4.0)	0.0259	0.40	0.3884	(0.17–0.93)
DQB1*06∶09	1 (0.3)	1 (0.1)	0.3673	3.90	NS	
DPB1*01∶01	1 (0.3)	0 (0.0)	0.2045	11.69	NS	
DPB1*02∶01	87 (24.0)	387 (27.5)	0.2061	0.83	NS	
DPB1*02∶02	15 (4.1)	59 (4.2)	1.0000	0.99	NS	
DPB1*03∶01	16 (4.4)	56 (4.0)	0.6573	1.12	NS	
DPB1*04∶01	10 (2.8)	55 (3.9)	0.3498	0.70	NS	
DPB1*04∶02	30 (8.3)	171 (12.1)	0.0409	0.65	0.6540	(0.44–0.98)
DPB1*05∶01	165 (45.6)	493 (35.0)	0.0002	1.55	0.0040	(1.23–1.96)
DPB1*06∶01	0 (0.0)	9 (0.6)	0.2181	0.20	NS	
DPB1*09∶01	24 (6.6)	112 (8.0)	0.4400	0.82	NS	
DPB1*13∶01	4 (1.1)	16 (1.1)	1.0000	0.97	NS	
DPB1*14∶01	5 (1.4)	25 (1.8)	0.8194	0.77	NS	
DPB1*17∶01	0 (0.0)	5 (0.4)	0.5900	0.35	NS	
DPB1*19∶01	2 (0.6)	5 (0.4)	0.6368	1.56	NS	
DPB1*38∶01	0 (0.0)	3 (0.2)	1.0000	0.55	NS	
DPB1*41∶01	1 (0.3)	7 (0.5)	1.0000	0.55	NS	
DPB1*47∶01	0 (0.0)	3 (0.2)	1.0000	0.55	NS	

RA: rheumatoid arthritis, Ro(+)La(−)RA: anti-Ro/SS-A-positive but anti-La/SS-B-negative RA, Ro(−)La(−)RA: ani-Ro/SS-A- and anti-La/SS-B-negative RA. OR: odds ratio, CI: confidence interval, *P*c: corrected *P* value, NS: not significant. Allele frequencies are shown in parenthesis (%). Associations were established by Fisher’s exact test using 2×2 contingency tables.

### Association of HLA Class II Allele Frequencies with the Presence of Anti-La/SS-B Antibodies

We then compared Ro(+)La(+)RA and Ro(−)La(−)RA *HLA class II* allele frequencies to seek associations with anti-La/SS-B antibodies. A significant positive association was found for DRB1*15∶01 and anti-La/SS-B antibodies (*Pc* = 0.0470, OR 3.14, 95%CI 1.63–6.05, Table 3). The DQB1*06∶02 allele was also associated with the presence of anti-La/SS-B antibodies (*Pc* = 0.0252, OR 3.14, 95% CI 1.63–6.05, Table 3). Further, the HLA-DPB1*05∶01 allele was also associated with anti-La/SS-B antibodies (*Pc* = 0.0069, OR 2.27, 95%CI 1.44–3.57, Table 3). Frequencies of SE and DR4 alleles were lower in Ro(+)La(+)RA than Ro(−)La(−)RA (*P* = 0.0367, OR 0.59, 95%CI 0.36–0.95, *P* = 0.0324, OR 0.57, 95%CI 0.34–0.95, respectively). Frequencies of DR2 alleles in Ro(+)La(+)RA were higher than Ro(−)La(−)RA patients (*P* = 0.0364, OR 1.81, 95%CI 1.06–3.09). Thus, there was an association of *HLA class II alleles* with anti-La/SS-B antibodies in RA patients.

**Table 3:** HLA allele frequencies in Ro(+)La(+) RA patients.

	Ro(+)La(+)	Ro(−)La(−)	*P*	OR	*Pc*	95%CI
DRB1*01∶01	3 (3.8)	110 (7.8)	0.2739	0.46	NS	
DRB1*03∶01	1 (1.3)	0 (0.0)	0.0538	53.15	NS	
DRB1*04∶01	0 (0.0)	52 (3.7)	0.1097	0.16	NS	
DRB1*04∶03	0 (0.0)	20 (1.4)	0.6214	0.42	NS	
DRB1*04∶04	0 (0.0)	3 (0.2)	1.0000	2.49	NS	
DRB1*04∶05	15 (18.8)	406 (28.8)	0.0555	0.57	NS	
DRB1*04∶06	1 (1.3)	25 (1.8)	1.0000	0.70	NS	
DRB1*04∶07	0 (0.0)	3 (0.2)	1.0000	2.49	NS	
DRB1*04∶10	5 (6.3)	33 (2.3)	0.0492	2.78	NS	(1.05–7.32)
DRB1*07∶01	1 (1.3)	6 (0.4)	0.3213	2.96	NS	
DRB1*08∶02	4 (5.0)	21 (1.5)	0.0414	3.48	NS	(1.16–10.38)
DRB1*08∶03	5 (6.3)	52 (3.7)	0.2280	1.74	NS	
DRB1*09∶01	6 (7.5)	243 (17.3)	0.0205	0.39	0.5728	(0.17–0.90)
DRB1*10∶01	0 (0.0)	12 (0.9)	1.0000	0.69	NS	
DRB1*11∶01	1 (1.3)	17 (1.2)	1.0000	1.04	NS	
DRB1*12∶01	4 (5.0)	39 (2.8)	0.2865	1.85	NS	
DRB1*12∶02	3 (3.8)	20 (1.4)	0.1223	2.70	NS	
DRB1*13∶01	0 (0.0)	3 (0.2)	1.0000	2.49	NS	
DRB1*13∶02	4 (5.0)	60 (4.3)	0.7735	1.18	NS	
DRB1*14∶02	0 (0.0)	2 (0.1)	1.0000	3.49	NS	
DRB1*14∶03	1 (1.3)	10 (0.7)	0.4566	1.77	NS	
DRB1*14∶05	2 (2.5)	14 (1.0)	0.2111	2.55	NS	
DRB1*14∶06	3 (3.8)	15 (1.1)	0.0681	3.62	NS	
DRB1*14∶07	0 (0.0)	1 (0.1)	1.0000	5.83	NS	
DRB1*14∶54	2 (2.5)	34 (2.4)	1.0000	1.04	NS	
DRB1*15∶01	12 (15.0)	75 (5.3)	0.0017	3.14	0.0470	(1.63–6.05)
DRB1*15∶02	7 (8.8)	124 (8.8)	1.0000	0.99	NS	
DRB1*16∶02	0 (0.0)	8 (0.6)	1.0000	1.02	NS	
DQB1*02∶01	2 (2.5)	6 (0.4)	0.0647	5.99	0.9710	
DQB1*03∶01	12 (15.0)	133 (9.4)	0.1183	1.69	NS	
DQB1*03∶02	4 (5.0)	73 (5.2)	1.0000	0.96	NS	
DQB1*03∶03	6 (7.5)	229 (16.3)	0.0392	0.42	0.5886	(0.18–0.97)
DQB1*03∶06	0 (0.0)	3 (0.2)	1.0000	2.49	NS	
DQB1*04∶01	15 (18.8)	432 (30.7)	0.0238	0.52	0.3573	(0.29–0.92)
DQB1*04∶02	6 (7.5)	35 (2.5)	0.0198	3.18	0.2974	(1.30–7.80)
DQB1*05∶01	3 (3.8)	125 (8.9)	0.1484	0.40	NS	
DQB1*05∶02	1 (1.3)	27 (1.9)	1.0000	0.65	NS	
DQB1*05∶03	3 (3.8)	29 (2.1)	0.2450	1.85	NS	
DQB1*06∶01	12 (15.0)	179 (12.7)	0.4962	1.21	NS	
DQB1*06∶02	12 (15.0)	75 (5.3)	0.0017	3.14	0.0252	(1.63–6.05)
DQB1*06∶03	0 (0.0)	3 (0.2)	1.0000	2.49	NS	
DQB1*06∶04	4 (5.0)	57 (4.0)	0.5656	1.25	NS	
DQB1*06∶09	0 (0.0)	1 (0.1)	1.0000	5.83	NS	
DPB1*02∶01	11 (13.8)	387 (27.5)	0.0061	0.42	0.0913	(0.22–0.80)
DPB1*02∶02	1 (1.3)	59 (4.2)	0.3713	0.29	NS	
DPB1*03∶01	5 (6.3)	56 (4.0)	0.3746	1.61	NS	
DPB1*04∶01	2 (2.5)	55 (3.9)	0.7656	0.63	NS	
DPB1*04∶02	4 (5.0)	171 (12.1)	0.0506	0.38	0.7591	
DPB1*05∶01	44 (55.0)	493 (35.0)	0.0005	2.27	0.0069	(1.44–3.57)
DPB1*06∶01	0 (0.0)	9 (0.6)	1.0000	0.92	NS	
DPB1*09∶01	6 (7.5)	112 (8.0)	1.0000	0.94	NS	
DPB1*13∶01	2 (2.5)	16 (1.1)	0.2517	2.23	NS	
DPB1*14∶01	4 (5.0)	25 (1.8)	0.0660	2.91	0.9907	
DPB1*17∶01	0 (0.0)	5 (0.4)	1.0000	1.58	NS	
DPB1*19∶01	0 (0.0)	5 (0.4)	1.0000	1.58	NS	
DPB1*38∶01	0 (0.0)	3 (0.2)	1.0000	2.49	NS	
DPB1*41∶01	0 (0.0)	7 (0.5)	1.0000	1.16	NS	
DPB1*47∶01	1 (1.3)	3 (0.2)	0.1985	5.93	NS	

RA: rheumatoid arthritis, Ro(+)La(+)RA: anti-Ro/SS-A- and anti-La/SS-B-positive RA, Ro(−)La(−)RA: anti-Ro/SS-A- and anti-La/SS-B-negative RA. OR: odds ratio, CI: confidence interval, *P*c: corrected *P* value, NS: not significant. Allele frequencies are shown in parenthesis (%). Associations were established by Fisher’s exact test using 2×2 contingency tables.

### Independent Associations of DRB1 and DPB1 with the Presence of Anti-Ro/SS-A Antibodies

A two-locus analysis was performed to identify the primary role of DRB1*08∶03, DQB1*06∶01, or DPB1*05∶01 for the production of anti-Ro/SS-A antibodies in RA patients. The OR for DRB1*08∶03 in patients lacking DQB1*06∶01 was 0.91 (not significant), while the OR for DQB1*06∶01 in patients without DRB1*08∶03 was 1.16 (not significant, Table 4). These differences did not reach statistical significance because of the strong linkage disequilibrium (LD) between DRB1*08∶03 and DQB1*06∶01 which results in a low frequency of DRB1*08∶03 in patients without DQB1*06∶01. On the other hand, the OR for DRB1*08∶03 in patients lacking DPB1*05∶01 was 5.59 (*Pc* = 0.0004), while it was 1.77 for DPB1*05∶01 in patients without DRB1*08∶03(*Pc* = 0.0002, Table 4). This suggests independent effects of DRB1*08∶03 and DPB1*05∶01 on the production of anti-Ro/SS-A antibodies in RA.

**Table 4:** HLA class II allele frequencies in RA cases with or without specific class II alleles.

	allele positivity		Ro(+)La(−)	Ro(−)La(−)	*P*	OR	*Pc*	95%CI
DRB1*08∶03	(−)	DQB1*06∶01	32 (10.9)	124 (9.5)	0.4481	1.16	NS	
	(−)	DPB1*05∶01	143 (48.6)	456 (34.9)	1.41×10^−5^	1.77	0.0002	(1.37–2.28)
	(+)	DQB1*06∶01	40 (58.8)	55 (53.9)	0.6364	1.22	NS	
	(+)	DPB1*05∶01	22 (32.4)	37 (36.3)	0.6254	0.84	NS	
DQB1*06∶01	(−)	DRB1*08∶03	0 (0.0)	2 (0.2)	1.0000	0.91	NS	
	(−)	DPB1*05∶01	119 (50.4)	421 (39.1)	0.0016	1.59	0.0264	(1.20–2.11)
	(+)	DRB1*08∶03	38 (30.2)	50 (15.2)	0.0005	2.42	0.0141	(1.49–3.93)
	(+)	DPB1*05∶01	46 (36.5)	72 (21.8)	0.0018	2.06	0.0294	(1.32–3.22)
DPB1*05∶01	(−)	DRB1*08∶03	14 (17.5)	22 (3.7)	1.41×10^−5^	5.59	0.0004	(2.73–11.45)
	(−)	DQB1*06∶01	23 (28.8)	111 (18.4)	0.0357	1.78	0.5356	(1.05–3.02)
	(+)	DRB1*08∶03	24 (8.5)	30 (3.7)	0.0023	2.41	0.0654	(1.38–4.19)
	(+)	DQB1*06∶01	49 (17.4)	68 (8.4)	0.0001	2.28	0.0011	(1.54–3.39)
	**allele positivity**		**Ro(+)La(+)**	**Ro(−)La(−)**	***P***	**OR**	***Pc***	**95%CI**
DRB1*15∶01	(−)	DQB1*06∶02	0 (0.0)	0 (0.0)				
	(−)	DPB1*05∶01	32 (53.3)	430 (34.0)	0.0033	2.22	0.0490	(1.32–3.74)
	(+)	DQB1*06∶02	12 (60.0)	75 (52.8)	0.6356	1.34	NS	
	(+)	DPB1*05∶01	12 (60.0)	63 (44.4)	0.2337	1.88	NS	
DQB1*06∶02	(−)	DRB1*15∶01	0 (0.0)	2 (0.2)	1.0000	4.19	NS	
	(−)	DPB1*05∶01	32 (53.3)	431 (33.9)	0.0033	2.22	0.0488	(1.32–3.74)
	(+)	DRB1*15∶01	12 (60.0)	73 (52.9)	0.6353	1.34	NS	
	(+)	DPB1*05∶01	12 (60.0)	62 (44.9)	0.2368	1.84	NS	
DPB1*05∶01	(−)	DRB1*15∶01	2 (20.0)	26 (4.3)	0.0724	5.54	NS	
	(−)	DQB1*06∶02	2 (20.0)	27 (4.5)	0.0771	5.32	NS	
	(+)	DRB1*15∶01	10 (14.3)	49 (6.1)	0.0205	2.57	0.5739	(1.24–5.34)
	(+)	DQB1*06∶02	10 (14.3)	48 (6.0)	0.0195	2.63	0.2918	(1.27–5.46)

RA: rheumatoid arthritis, Ro(+)La(−)RA: anti-Ro/SS-A-positive but anti-La/SS-B-negative RA, Ro(−)La(−)RA: anti-Ro/SS-A- and anti-La/SS-B-negative RA, Ro(+)La(+)RA: anti-Ro/SS-A- and anti-La/SS-B-positive RA, OR: odds ratio, CI: confidence interval, *P*c: corrected *P* value, NS: not significant. Allele frequencies are shown in parenthesis (%). Associations were established by Fisher’s exact test using 2×2 contingency tables.

The two-locus analysis was also performed to identify the primary role of DRB1*15∶01, DQB1*06∶02, or DPB1*05∶01 for the production of anti-La/SS-B antibodies in RA patients. OR for DPB1*05∶01 was 2.22 in patients without DRB1*15∶01 (*Pc* = 0.0490, Table 4) and was 2.22 in patients without DQB1*06∶02 (*Pc* = 0.0490, Table 4). This might suggests independent effects of DPB1*05∶01 and DRB1*15∶01 or DQB1*06∶02 on the production of anti-La/SS-B antibodies in RA.

### Effects of SE on the Association of HLA Class II Alleles

We examined whether the positive association of DRB1*08∶03, DQB1*06∶01, and DPB1*05∶01 alleles is secondary to the decrease of SE in the Ro(+)La(−)RA patients. When the patients with SE were excluded from the analysis, the DRB1*08∶03 and DQB1*06∶01 allele frequencies were significantly higher in Ro(+)La(−)RA than Ro(−)La(−)RA (*Pc* = 0.0008, OR 3.33, 95%CI 1.91–5.82, and *Pc* = 0.0191, OR 2.05, 95%CI 1.33–3.15, respectively, Table 5). On the other hand, DPB1*05∶01 frequency was still higher in Ro(+)La(−)RA than Ro(−)La(−)RA, although the effect was not statistically significant (*Pc* = 0.5887, OR 1.53, 95%CI 1.03–2.28, Table 5).

**Table 5:** HLA class II allele frequency in the RA cases without SE alleles.

	Ro(+)La(−)	Ro(−)La(−)	*P*	OR	*Pc*	95%CI
DRB1*0803	29 (21.0)	29 (7.4)	4.60×10^−5^	3.33	0.0008	(1.91–5.82)
DQB1*0601	47 (34.1)	79 (20.2)	0.0016	2.05	0.0191	(1.33–3.15)
DPB1*0501	60 (43.5)	131 (33.4)	0.0392	1.53	0.5887	(1.03–2.28)
	**Ro(+)La(+)**	**Ro(−)La(−)**	***P***	**OR**	***Pc***	**95%CI**
DRB1*1501	7 (20.6)	36 (9.2)	0.0660	2.56	NS	
DQB1*0602	7 (20.6)	36 (9.2)	0.0660	2.56	0.7915	
DPB1*0501	15 (44.1)	131 (33.4)	0.2579	1.57	NS	

SE: shared epitope, OR: odds ratio, CI: confidence interval, *Pc*: corrected *P* value, NS: not significant. Allele frequencies are shown in parenthesis (%). Associations were established by Fisher’s exact test using 2×2 contingency tables.

We also examined whether the positive association of DRB1*15∶01, DQB1*06∶02, and DPB1*05∶01 alleles is secondary to the decrease of SE in the Ro(+)La(+)RA patients. When the patients with SE were excluded from the analysis, these allele frequencies were still higher in the Ro(+)La(+)RA than Ro(−)La(−)RA, although the effect was not statistically significant (Table 5).

### Effects of Secondary SS on the Association of HLA Class II Alleles

The associations of anti-Ro/SS-A and anti-La/SS-B antibodies with secondary SS were described in Table 1, and the association of secondary SS with HLA class II alleles was investigated. No association was observed between DPB1*05∶01 or anti-Ro/La antibody-associated HLA alleles and secondary SS. The associations of DRB1*08∶03 (*P*c = 0.0001, OR 3.13, 95%CI 1.97–4.96), DQB1*06∶01 (*P*c = 0.0173, OR 1.75, 95%CI 1.27–2.43), or DPB1*05∶01 (*P*c = 0.0064, OR 1.59, 95%CI 1.23–2.05) with anti-Ro/SS-A antibodies remain significant after excluding RA patients with secondary SS. However, the associations of DRB1*15∶01 (*P*c = 0.3450, OR 2.93, 95%CI 1.34–6.42), DQB1*06∶02 (*P*c = 0.1848, OR 2.93, 95%CI 1.34–6.42), or DPB1*05∶01 (*P*c = 0.0941, OR 2.16, 95%CI 1.26–3.70) with anti-La/SS-A antibodies did not reach statistical significance after excluding RA patients with secondary SS, because of reduced sample numbers after the exclusion.

### Certain Amino Acid Residues in the DRβ, DQβ, and DPβ Chains are Associated with the Presence of Anti-Ro/SS-A Antibodies

**Figure 1:**
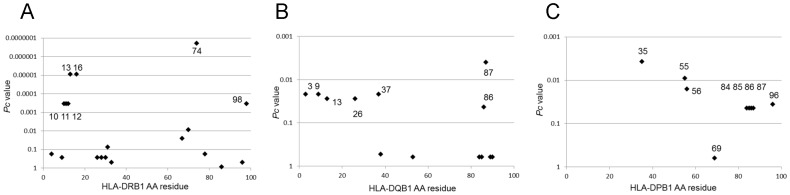
Associations of amino acid residues in DRβ (A), DQβ (B), and DPβ chains (C) with the presence of anti-Ro/SS-A antibodies. Corrected *P* (*P*c) values were calculated by multiplying the *P* value by the number of amino acid residues tested. Associations were established by Fisher’s exact test using 2×2 contingency tables.

Amino acid residues in HLA-DRβ, DQβ, and DPβ chains were analyzed for their potential associations with anti-Ro/SS-A antibodies. Amino acid positions 13, 16, and 74 in the DRβ chain showed strong associations with the presence of anti-Ro/SS-A antibodies (Figure 1A). The amino acid residue at position 74 associated with anti-Ro/SS-A is different from the SE at that position; these three amino acid residues (13, 16 and 74) are shared by DRB1*08∶02, *08∶03, and *08∶23. The amino acid position 87 in the DQβ chain showed associations with anti-Ro/SS-A antibodies (Figure 1B). Finally, amino acid positions 35, 55, and 56 in the DPβ chain showed associations with anti-Ro/SS-A antibodies (Figure 1C). These three residues are shared by DPB1*05∶01 and *38∶01. Thus, certain amino acid residues in the DRβ, DQβ, and DPβ chains are associated with the presence of anti-Ro/SS-A antibodies.

### Certain Amino Acid Residues in the DRβ, DQβ, and DPβ Chains are Associated with the Presence of Anti-La/SS-B Antibodies

**Figure 2:**
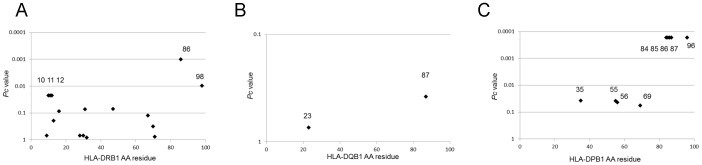
Associations of amino acid residues in DRβ (A), DQβ (B), and DPβ chains (C) with the presence of anti-La/SS-B antibodies. Corrected *P* (*P*c) values were calculated by multiplying the *P* value by the number of amino acid residues tested. Associations were established by Fisher’s exact test using 2×2 contingency tables.

Amino acid residues in HLA-DRβ, DQβ, and DPβ chains were also analyzed for associations with anti-La/SS-B antibodies. Positions 86 in the DRβ chain showed strong associations with anti-La/SS-B antibodies (Figure 2A). No associations were observed for any residues in the DQβ chain (Figure 2B), whereas positions 84–87 and 96 in the DPβ chain showed associations with anti-La/SS-B antibodies (Figure 2C). Thus, association analysis suggested roles for certain defined amino acid residues in DRβ and DPβ.

### Association of HLA-DPB1*05∶01 with the Presence of Anti-Ro/SS-A and Anti-La/SS-B Antibodies in SLE Patients

We also tested whether *HLA-DPB1* was associated with the presence of anti-Ro/SS-A or anti-La/SS-B antibodies in SLE patients. The DPB1*05∶01 allele was associated with anti-Ro/SS-A (*Pc* = 0.0408, OR 1.69, 95% CI 1.19–2.41, Table 6) and anti-La/SS-B antibodies (*Pc* = 2.48×10^−5^, OR 3.31, 95%CI 2.02–5.43, Table 6). A significant negative association was found for DPB1*02∶01 and anti-La/SS-B antibodies (*Pc* = 0.0283, OR 0.34, 95%CI 0.17–0.70, Table 6). Thus, there was an association of DPB1*05∶01 allele with anti-Ro/SS-A and anti-La/SS-B antibodies in SLE patients.

**Table 6:** *HLA-DPB1* allele frequency in the SLE patients.

				Ro(+)La(−) vs. Ro(−)La(−)				Ro(+)La(+) vs. Ro(−)La(−)			
	Ro(+)La(−)	Ro(+)La(+)	Ro(−)La(−)	P	OR	*Pc*	95%CI	P	OR	*Pc*	95%CI
DPB1*01∶01	0 (0.0)	1 (1.1)	0 (0.0)					0.2473	9.20	NS	
DPB1*02∶01	49 (19.0)	10 (11.1)	73 (26.6)	0.0393	0.65	0.4711	(0.43–0.97)	0.0022	0.34	0.0283	(0.17–0.70)
DPB1*02∶02	11 (4.3)	3 (3.3)	19 (6.9)	0.1939	0.60	NS		0.3084	0.46	NS	
DPB1*03∶01	13 (5.0)	3 (3.3)	10 (3.6)	0.5239	1.40	NS		1.0000	0.91	NS	
DPB1*04∶01	6 (2.3)	0 (0.0)	9 (3.3)	0.6045	0.70	NS		0.1198	0.15	NS	
DPB1*04∶02	27 (10.5)	7 (7.8)	25 (9.1)	0.6622	1.16	NS		0.8315	0.84	NS	
DPB1*05∶01	118 (45.7)	56 (62.2)	91 (33.2)	0.0034	1.69	0.0408	(1.19–2.41)	1.91×10^−6^	3.31	2.48×10^−5^	(2.02–5.43)
DPB1*06∶01	1 (0.4)	0 (0.0)	2 (0.7)	1.0000	0.53	NS		1.0000	0.60	NS	
DPB1*09∶01	18 (7.0)	4 (4.4)	23 (8.4)	0.6264	0.82	NS		0.2544	0.51	NS	
DPB1*13∶01	6 (2.3)	3 (3.3)	10 (3.6)	0.4509	0.63	NS		1.0000	0.91	NS	
DPB1*14∶01	8 (3.1)	2 (2.2)	8 (2.9)	1.0000	1.06	NS		1.0000	0.76	NS	
DPB1*19∶01	1 (0.4)	0 (0.0)	2 (0.7)	1.0000	0.53	NS		1.0000	0.60	NS	
DPB1*38∶01	0 (0.0)	0 (0.0)	2 (0.7)	0.4995	0.21	NS		1.0000	0.60	NS	

SLE: systemic lupus erythematosus, Ro(+)La(−): anti-Ro/SS-A-positive but anti-La/SS-B-negative, Ro(+)La(+): anti-Ro/SS-A- and anti-La/SS-B-positive, Ro(−)La(−): anti-Ro/SS-A- and anti-La/SS-B-negative SLE patients. OR: odds ratio, CI: confidence interval, *Pc*: corrected *P* value, NS: not significant. Allele frequencies are shown in parenthesis (%). Associations were established by Fisher’s exact test using 2×2 contingency tables.

## Discussion

Several studies have shown that certain *HLA-DR* alleles are associated with the presence of anti-Ro/SS-A or anti-La/SS-B antibodies in patients with autoimmune diseases. However, few studies have focused on the association of HLA alleles with anti-Ro/SS-A or anti-La/SS-B antibodies in RA. To the best of our knowledge, this is the first report of a positive association of HLA-DPB1*05∶01 with anti-Ro/SS-A and anti-La/SS-B antibodies in RA, although a tendency towards a higher frequency of this allele in Japanese patients with anti-Ro/SS-A or anti-La/SS-B antibodies has been reported before [9]. Recent studies have noted associations of *DPB1* alleles with several diseases [16], [17], [18], [19], [20], but here we report an association of DPB1*05∶01 with anti-Ro/SS-A and anti-La/SS-B antibodies in Japanese RA and SLE patients.

It was reported that RA patients with anti-Ro/SS-A antibodies had a more severe disease course, and that they were less frequently DR4-positive than patients without such antibodies [21], [22]. In contrast, the presence of anti-Ro/SS-A antibodies in RA was reported to be positively associated with DR4 in some other studies [10], [23]. Here, we found that frequencies of DR4 and the SE were lower in anti-Ro/SS-A-positive RA patients (Figure 2). Although the implications of this finding are not clear, it might suggest that the role of SE may not be as important in anti-Ro/SS-A-positive RA. Alternatively, the genetic background of anti-Ro/SS-A-positive and -negative RA may be different, and genetic factors other than SE may play a significant role in the former.

It was reported that anti-Ro/SS-A-positive patients were more frequently DR3- or DR2-positive in the context of other autoimmune diseases like primary SS and SLE in European populations [5]. An association of DRB1*15∶01 and anti-Ro/SS-A antibodies has been reported in the Japanese population [9]. Such an association of DR2 with the presence of anti-Ro/SS-A was not confirmed in our study on RA patients, but an association of DR2 with the presence of anti-La/SS-B was observed. Although DRB1*08∶03 was reported to be associated with anti-La/SS-B in Japanese [9], we observed here that it was associated with the presence of anti-Ro/SS-A antibodies in our RA patients. These could be explained by differences in the pathogenesis of RA and SLE.

Amino acid residues 13, 16 and 74 of the HLA-DRβ chain were found to be associated with the presence of anti-Ro/SS-A antibodies (Figure 1). Residues 13 and 74 form the HLA-DR peptide-binding groove [24]. Amino acid residues 84–87 and 96 in the DPβ chain were associated with anti-La/SS-B antibodies. Similarly, amino acid residues 85 and 86 form the peptide-binding grooves of HLA-DP molecules. These data suggest the involvement of peptide antigens bound to specific HLA molecules in controlling the production of anti-Ro/SS-A or anti-La/SS-B antibodies.

It has been determined that patients with anti-Ro/SS-A antibodies are more prone to develop adverse effects when treated with gold salts and other drugs [25], [26], [27]. Recent studies have shown that adverse drug reactions are associated with drug-specific *HLA class I* alleles, for example A*31∶01 and B*15∶02 with carbamazepine, and A*31∶01 with methotrexate [28], [29], [30]. Furthermore, A*31∶01 is in LD with DRB1*08∶03 and *15∶01 (0.56% and 0.63% of haplotype frequency, respectively, see http://hla.or.jp/haplo/haplonavi.php?type=haplo&lang=en). Frequencies of these class II alleles were higher in anti-Ro/SS-A or anti-La/SS-B antibody- positive RA patients (Tables 2, 3). Large scale association studies for *HLA class I* and anti-Ro/SS-A or anti-La/SS-B antibodies should be performed. Because of the limited sample size of the present study, the observed significance of the statistical association was modest. The association with *HLA-DP* needs to be confirmed in future independent studies. Because the allelic distribution of *HLA* in other ethnic populations is different from that in the Japanese, the role of *HLA-DP* in anti-Ro/SS-A or anti-La/SS-B antibody production in RA in other populations should be determined.

This is the first identification of an association of HLA-DPB1*05∶01 with positivity for anti-Ro/SS-A or anti-La/SS-B antibodies in RA. Our findings support the role of *HLA-DP*, as well as *DR,* in the pathogenesis of autoantibody production.
